# A Quantification Method for Disorganized Bone Components: Application to the Femoral Shaft

**DOI:** 10.1002/jbm4.10713

**Published:** 2023-01-03

**Authors:** Roger Zebaze, Catherine Shore‐Lorenti, Hanh H Nguyen, Cherie Chiang, Frances Milat, Peter R Ebeling

**Affiliations:** ^1^ Department of Medicine School of Clinical Sciences, Monash University Clayton Victoria Australia; ^2^ Department of Endocrinology Monash Health Clayton Victoria Australia; ^3^ Austin Health, Department of Medicine University of Melbourne Heidelberg Victoria Australia; ^4^ Hudson Institute of Medical Research Clayton Victoria Australia

**Keywords:** Accuracy and precision, Atypical femoral fracture, Disorders of disorganizedbone tissue, Ineffective load conduction or transfer, Quantification ofdisorganized bone

## Abstract

Based on the current paradigm, a healthy bone is one with adequate mass without microarchitectural decay. However, these two features may not be sufficient to ensure that a bone is healthy. In addition, components must be correctly assembled and aligned. This ensures “*the right amount of bone, at the right place*” and thus, an optimal cohesion or interplay between constituents. Disorganization may be an independent contributor to bone abnormalities including fragility fractures. Indeed, many bone diseases may be characterized by the presence of disorganized bone, including osteogenesis imperfecta, hypophosphatasia, and atypical femur fractures (AFFs). Despite its likely importance, currently, there are no tools to quantify disorganization in vivo. We address this unmet need by describing a novel method for quantifying bone disorganization from X‐ray images. Disorganization is quantified as variations in the orientation of bone components in relation to a target reference point. True disorganization created by disarranging (misplacing) pixels within the bone served as “gold standard.” To further validate the method in clinical settings, we compared disorganization in three groups of femurs: (i) femurs of women with AFFs (*n* = 9); (ii) fracture‐free femurs contralateral to AFFs (*n* = 9); and (iii) fracture‐free femurs from controls (*n* = 25). There was excellent agreement between measured disorganization and “gold standard,” with *R* ^2^ values ranging from 0.84 to 0.99. Precision error ranged from 1.72% to 4.69%. Disorganization produced by abnormalities associated with AFFs was accurately captured. Disorganization level was lowest in fracture‐free control femurs, higher in fracture‐free contralateral femurs to AFFs, and highest in femurs with AFFs (all *p* < 0.0001). Quantification of disorganization, a novel biomarker, may provide novel insights into the pathogenesis of metabolic bone diseases beyond that provided by bone mineral density (BMD) or microarchitecture. We provide evidence that measurement of disorganization is likely to help identify patients at risk for fractures, especially in those poorly explained by BMD or microarchitecture such as AFFs. © 2023 The Authors. *JBMR Plus* published by Wiley Periodicals LLC on behalf of American Society for Bone and Mineral Research.

## Introduction

A major challenge in the field of bone health is that most fractures (>75%) occur in patients who do not have osteoporosis (defined by a bone mineral density [BMD] *T*‐score ≤ −2.5).^(^ ^1^ ^)^ It was initially believed that these fractures are due to microstructural deterioration—an abnormality that may be present but missed by BMD measurement, which is a two‐dimensional (2D) projection of a three‐dimensional (3D) structure.^(^ ^2^ ^)^ To address this, many tools have been developed to quantify bone microarchitecture and features of structural decay (porosity, thinning, loss of connectivity). These tools include quantitative computed tomography (QCT) or high‐resolution peripheral QCT (HR‐pQCT).^(^ ^2^, ^3^ ^)^ Indirect approaches such as trabecular bone score (TBS) are now also used. In addition, to better estimate bone strength, and hence fracture risk, many tools such as finite element analysis (FEA) or microindentation testing have been implemented.^(^ ^2^, ^3^ ^)^ These novel advances have undoubtedly helped identify many more patients who sustain fragility fractures but do not have osteoporosis on bone densitometry.^(^ ^4^ ^)^ However, many such patients sustaining fragility fractures remain unidentified despite their use.^(^ ^4^, ^5^ ^)^

According to the current paradigm, a healthy bone is one with adequate amount of mineralized matrix providing normal BMD.^(^ ^6^ ^)^ Furthermore, the components (structural elements) forming this bone must be “healthy”; ie, without microarchitectural deterioration.^(^ ^4^ ^)^ A bone that has these two properties is viewed according to the prevailing paradigm as healthy and at little to no risk for fragility fractures. Treatment‐related changes in BMD are also now viewed as a surrogate biomarker for fracture risk reduction.^(^ ^7^ ^)^ However, this current view has still many limitations, as highlighted by the occurrence of fractures in patients with normal to high bone mass, no decay, and normal to increased bone strength.^(^ ^8^ ^)^ Atypical femoral fractures (AFFs) are stress fractures showing little to no association with BMD, bone architecture, or strength.^(^ ^5^, ^9^ ^)^ This, therefore, suggests that there are other bone properties and abnormalities that may predispose to fragility fractures but remain unquantified.

We propose that even if a bone has a normal to high BMD and no structural decay, it does not necessarily mean that this bone is healthy. In addition to these properties, the components (or structural elements) forming the bone must be properly assembled and aligned. This ensures “*the right amount of bone, at the right place*” and hence, an optimal cohesion or interplay between bone components. The lack of a correct assembly of components—ie, bone disorganization—is an anomaly (aberration); a likely key independent contributor to bone abnormalities including fragility fractures. Indeed, many bone diseases are characterized by the presence of disorganized bone. Notable examples include osteogenesis imperfecta (OI), hypophosphatasia, osteopetrosis, chronic kidney disease metabolic bone disorder (CKD‐MBD), diabetes mellitus, and antiresorptive therapy‐associated AFFs.^(^ ^9^, ^10^, ^11^, ^12^, ^13^, ^14^ ^)^ Therefore, a comprehensive assessment of these diseases requires identification and quantification of the extent of associated bone disorganization.

Despite the likely critical role of disorganization in bone diseases, there are currently no tools for the measurement of this feature in vivo.

Hence, to address this important unmet need, we hereby report an accurate and reproducible novel method for detecting and quantifying the extent of bone disorganization from X‐ray images. The method measures the extent to which pixels forming a bone are disorganized both in their respective locations, and attenuation values. Pixels attenuation reflect, and is a good surrogate of local bone tissue composition. This forms the basis of using X‐ray imaging to assess bone tissues at various lengths of scales (soft to hard) and identify abnormalities.^(^ ^15^ ^)^

The method is described here more particularly, in the context of disorganized bone within the femoral shaft. Disorganization is measured on standard routine pelvic X‐ray images extending to the femoral shaft. The femur was chosen because this is the site of AFFs.

## Materials and Methods

### Data source/participants/images

Because the method is described here is in the context of the femoral shaft, images are derived from standard pelvic or hip X‐ray images. The radiation dose is low: <0.7 mSv/measurement.^(^ ^16^ ^)^

Participants were recruited as described.^(^ ^17^ ^)^ This study includes all individuals aged ≥50 years who were admitted to Monash Health with femoral fractures. They were identified via the hospital's electronic database. Data pertaining to each study member were electronically extracted from the database. Participants with AFFs are also part of the Transcontinental Atypical Femoral Fracture Consortium (TRAFFIC) study. This is an international consortium investigating the risk factors and identifying genetic causes and ways to prevent AFFs. The following participants were included in this study:

#### Fracture‐free participants

A total of 35 women were distributed as follows:

- *One* ^(^ ^1^ ^)^ *participant* was used for in vitro experimental simulation. From the femoral image, seven different patterns of disorganization were simulated with three different attenuation values giving a total of 21 femoral images.
  - The first experiment involved a simulation based on the attenuation value of zero (0). This was selected to test the ability of the method to accurately quantify disorganization produced by soft tissue.
  - The second experiment involved simulation based on the attenuation value of 165. This represents a pixel comprising both soft and mineralized tissue.
  - The last experiment involved the attenuation value of 220 to simulate disorganization due to pixels comprising mainly mineralized bone matrix.
- *Nine participants*—were recruited for reproducibility studies.
- *25 participants—*were recruited as controls for further in vivo clinical validation.

#### Participants with AFFs

A total of 12 women were recruited.

- Nine participants were recruited on the basis of having within the same image both an AFF and a fracture‐free contralateral site. This was useful for comparison and validation.
- Three participants were recruited for exemplary illustration of the quantitative method.

The study was approved by the Monash Health Human Research Ethics Committee.

Images were processed using a novel customized software (OAS_1.0_, Melbourne, Australia) to detect and quantify disorganization (misalignment, disarrangement) between bone components.

### Segmentation of bone from the external environment

**Fig. 1:**
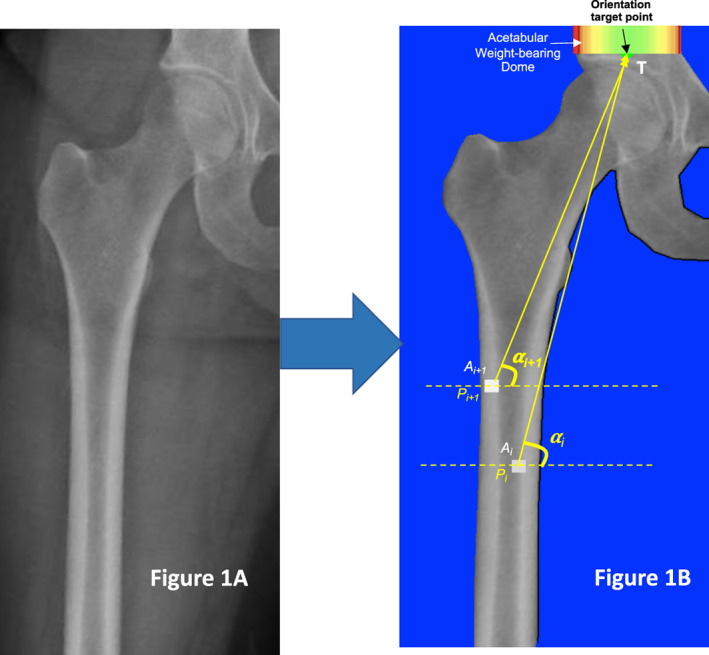
(*A*) An example of images used for the quantification of disorganization. An original X‐ray image of left pelvis with a focus on the femoral shaft is shown. (*B*) Illustration of the process of pixel‐by‐pixel quantification of disorganization. Nonbone tissue has been segmented and color‐coded blue. The acetabular weight‐bearing dome is color‐coded in spectrum from green to red. The central part of the dome is colored green. The middle which is the orientation target point (T) is shown by the black arrow. The angle between the vector (α_*i*_) defined by the Pixel (P_*i*_) and the Target referent point $\left(\underset{P_{i}T_{\mathrm{rp}}}{\to }\right)$ is calculated. Then, the angle between the vector (α_*i*+1_) defined by another Pixel (P_*i*+1_) and the Target referent point $\left(\underset{P_{i+1}T_{\mathrm{rp}}}{\to }\right)$ is calculated. The Disorganization value between Pi and P_*i*_ + 1 (DV_*i*, *i*+1_) is calculated as the attenuation weighted absolute difference in the orientation of the two vectors. ${\mathrm{DV}}_{i,i+1}=\mathrm{ABS}\left(A_{i}\times \alpha_{i}-A_{i+1}\times \alpha_{i+1}\right)$.

The process starts with the delineation of the periosteal contours so that mineralized bone matrix can be separated (segmented) from nonbone tissue. Digital segmentation of the bone from surrounding soft tissue was performed by global thresholding. The global threshold for each sample in the dataset was determined by selecting a local minimum in the frequency plot of pixel gray levels for that sample.^(^ ^18^ ^)^ Manual correction of contours was performed if needed (Fig. 1). For the quantification of disorganization, no segmentation of bone into compartments (cortical, trabecular, or transitional zone) is required and none was performed.

### Quantification of disorganized (misassembled, disarranged, misaligned) bone tissue

This involved a pixel‐by‐pixel analysis of the segmented bone by an in‐house custom software (OAS_1.0_, Melbourne, Australia). The device measures the orientation (direction, alignment) of each pixel (*P*_*i*_) vis‐à‐vis the upper body (represented by the middle of the acetabular weight‐bearing dome) (Fig. 1). This referent target point was chosen because loads from the upper body enter the femur via the acetabular weight‐bearing dome.^(^ ^19^ ^)^ Disorganization—ie, misarrangement of pixel (*P*_*i*_) relative to pixel (*P*_*i+*1_)—is then quantified as the difference (or deviation) in their orientation vis‐à‐vis this reference target point. To account for not only the spatial heterogeneity but also the composition, the disorganization so calculated was further weighted by the attenuation of each pixel.

If all pixels have the same orientation in relation to the target point then, local bone components are well‐organized, arranged. Conversely, the greater the differences in the orientations of pixels vis‐à‐vis the target point, the greater the misarrangement or disorganization between these pixels. For each level (section) of the femur, the average disorganization is computed. Finally, from the measured disorganization values, the software (OAS_1.0_) outputs for each image, a unique customized Disorganization Quantifier APP (Alignogram) that displays the extent of disorganization (misalignment, disarrangement) of bone at each location along the femur.

### Accuracy of disorganization (misaligned, disarranged) of bone tissue

#### In vitro experimental simulations

A disorganized component is an element located where it should not be. Hence, to assess the accuracy of disorganization quantification, in an X‐ray image of the femur, at a given location, pixels forming the bone were disarranged, misplaced.

Misplaced pixels were selected in such a way that their attenuation values reflect various types of bone tissues: soft tissues, a mixture of soft and hard tissue, and mineralized or hard tissue.

For each of the three types of simulated bone tissues, seven different patterns of disorganization were generated given, a total of 21 images. Each image was then analyzed using the OAS_1.0_ software to quantify the so‐created disorganization.

- *The first experiment* involved simulations based on the attenuation values of zero (0). This value was selected to test the ability of the method to accurately quantify disorganization produced by soft tissue. Such attenuation values are observed in pixels that contain only soft tissues and no mineralized bone matrix.^(^ ^20^ ^)^
- *The second experiment* involved simulations based on attenuation of values 165. This represents a pixel comprising both soft and mineralized matrix at different stages of completeness of secondary mineralization.^(^ ^21^ ^)^
- *The third experiment* involved attenuation values of 220 to simulate disorganization due to pixels comprising mainly or fully mineralized bone tissue. This value of 220 corresponds to 86% of the maximum grayscale (ie, 255). Pixels containing bone matrix undergoing secondary mineralization have an attenuation value equal to or greater than 80% of the maximum.^(^ ^21^, ^22^, ^23^ ^)^

The following disorganization parameters caused by the disarrangement (disarray) of pixels were measured:

- A graph (denoted ALIGNOGRAM) that displays the extent of disorganization (or disarrangement) of bone pixels at each position along the femur.
- Statistical measures of disorganization from the curve (ALIGNOGRAM). This is done over the entire femur, or a portion of it and includes:
  - The mean disorganization value (MDV).
  - The standard deviation of all disorganization values (SDDV).
  - The critical disorganization value (CDV): the threshold value about which any disorganization value (DV) is an outlier (ie, critical). This is calculated as disorganization values above 1.5 interquartile range (IQR) above the 3rd quartile (Q3) for all DVs displayed on the ALIGNOGRAM curve.
- In addition to the absolute disorganization values at each location along the femur, areas of with disorganization levels higher than expected were also detected. To do so, at each location along the femur, a peak disorganization value (PDV) was calculated as 100* DV/MDV, where DV is the disorganization value at the location and MDV is the mean disorganization value for the whole femur displayed on the curve. From these PDV, the following metrics were computed:
  - The average or mean PDV for the entire femur (or a portion of it)
  - The maximum PDV disorganization for the entire femur (or a portion of it)
- The location of the maximum PDV was detected and displayed on the ALIGNOGRAM.

Agreements between the extent of bone disorganization created by mispositioning pixels (“gold standard”) (true disorganization) versus disorganization metrics measured by the device were assessed.

#### In vivo clinical verification/validation

For clinical validation, disorganization was quantified in femurs of women with AFFs and in those of fracture‐free age matched controls. Various stages of bone abnormalities ranging from periosteal and/or endosteal reactions (with or without cortical thickening), dreaded‐black line, incomplete to complete AFFs were assessed. Disorganization was measured in the femora, curves (ALIGNOGRAMs) were outputted and metrics of bone disorganization were calculated. The extent of X‐ray abnormalities present (as assessed by experts' visual assessment RZ and PRE) was correlated with the measured bone disorganization. Atypical femoral fractures were diagnosed based on criteria defined by the 2014 American Society for Bone and Mineral Research Task Force Report.^(^ ^9^ ^)^

To further validate the performance of the method in clinical settings, we compared disorganization values in three groups of women: (i) *Femurs of women with AFFs* (*n* = 9). These are bones with demonstrably high to extreme levels of disorganization (misalignment, misplacement) of bone components. (ii) *Fracture‐free contralateral femur to the one with AFFs* (*n* = 9). It is reasonable to assume that these femurs have higher levels of disorganization than controls. However, these values are not severe enough to reach levels of contralateral AFFs sites. (iii) *Fracture‐free femurs from age‐matched women* (*controls*) (*n* = 25). It is reasonable to assume these femurs do not have any significant disorganization. Hence, disorganization in these femurs (if any), should be lower than that observed in fracture‐free contralateral femurs to AFFs, and lower than the disorganization levels observed in AFF sites.

This clinical experiment allowed us to assess the ability of the method to identify patients at risk of AFFs.

##### Precision

This involved repeating the process of quantification of bone disorganization three times in nine women.

### Statistical analysis

Data were analyzed and expressed in absolute terms, percentage differences, standard deviations (SDs), and coefficients of variation (SD/mean). To validate the quantification of disorganization, linear regressions were used to determine the *R* ^2^ between the gold standard (true disorganization) and disorganization measured using the method. Bland‐Altman plots were used to assess differences between any two measurements. Differences in disorganizations between fracture‐free femurs and those with AFFs were shown as box‐and‐whisker plots. We used the nonparametric Mann‐Whitney *U*‐test for comparisons. The precision error was calculated as the root mean square (RMS) of the coefficients of variation (CV%). Significance levels (*p* < 0.05) were two‐tailed.

## Results

### Accuracy of disorganization quantification—in vitro experimental simulations

**Fig. 2:**
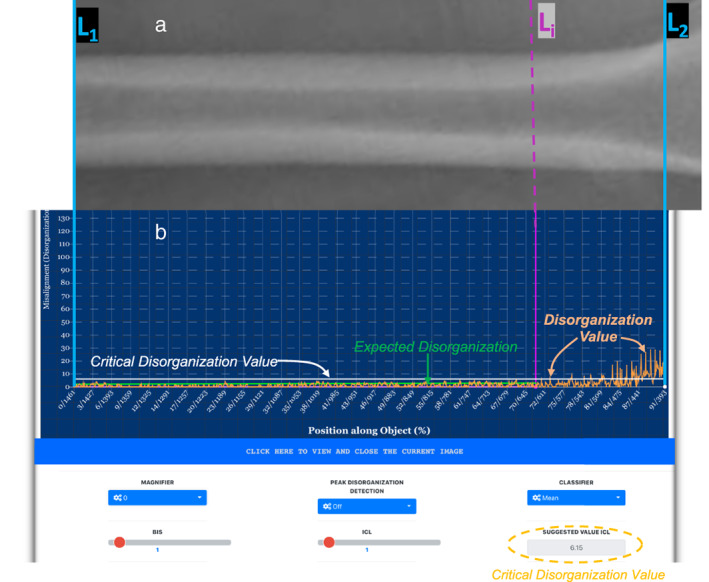
(*A*) Top panel shows an X‐ray image of the femoral shaft of a healthy woman. (*B*) Shows the screen of the quantifier (blue background) with the dashboard below. On the *x*‐axis is the position along the femur starting with the highest ranking (most distal portion‐L1) and the lowest ranking being the lesser trochanter (L2). On the *y*‐axis, is the quantified disorganization (misarrangement, misalignment) values. The orange curve shows the pattern of DVs along the shaft. The green line shows the expected disorganization values between the most distal portion of the shaft and any other location within the femur (Li‐purple line). The white line is the threshold value above which any disorganization value between L1 and Li is considered an outlier. In a normal person, at the distal end, and going up to near the lesser trochanter, the disorganization curve is relatively flat, regular with low DVs. In this example, DVs are lower than 10 (arbitrary unit). Few are above the green lines, and none exceeds the white line. Close to the lesser trochanter, a steady and gradual increase in DVs. The upsloping of the curve matches the gradual, physiological transition and adjoining of the shaft with the neck. At the level of the lesser trochanter disorganization values are much higher reaching values of 30 (arbitrary units). DV = disorganization value.

Figure 2*A* is an X‐ray image of the femur of a healthy postmenopausal woman. Disorganization of the same femoral shaft as measured is displayed on the graph (ALIGNOGRAM) in Fig. 2*B*. The measurements are performed along the entire femoral shaft from the distal end (Location‐L_1_) to the lesser trochanter (Location‐L_2_) (Fig. 2, cyan lines). The analysis also can be performed on a subportion of the femoral shaft between the distal end of the femoral shaft (L_1_) and any other location (L_*i*_) (Fig. 2, from L_1_[cyan line] to L_*i*_ [purple LINE]).

In Fig. 2*B*, on the *x*‐axis is the location along the femoral shaft (in pixel ranking) with the highest rank assigned to the most distal location. On the *y*‐axis is the DV (in arbitrary unit) at each location (*x*) along the shaft in pixels ranking (and percent length of the shaft). The expected disorganization values between the distal end of the shaft (L_1_) and any location (L_*i*_) is measured and displayed as a green line. Any DV above CDV (*white line* on the graph) is an outlier. The numerical value of the CDV is shown (Fig. 2, yellow dotted circle).

The orange curve shows the DVs at each position along the shaft. As shown in Fig. 2*B*, the extent of bone disorganization (disarrangement, misalignment) along the distal end, and up to close to the lesser trochanter is small. As a result, the curve is relatively regular, nearly flat, with minimal to no abrupt or striking peaks. However, the degree of disorganization increases gradually, but steadily at more proximal femoral shaft locations close to the lesser trochanter. The extent of disorganization at the lesser trochanter is markedly higher than at the femoral shaft (Fig. 2*B*).

**Fig. 3:**
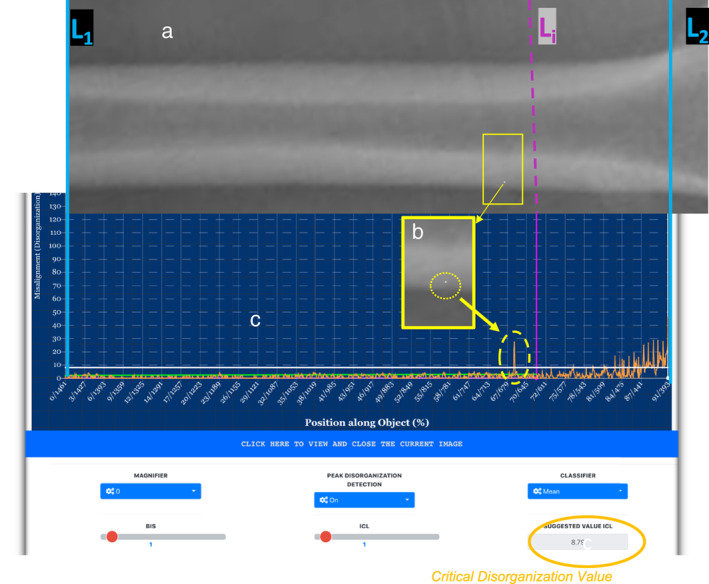
(*A*) is the same femur in Fig. 2*A* (healthy women). In this femur, a single pixel of attenuation value 220 has been misarranged to produce disorganization (a whiter pixel in the yellow rectangle). (*B*) Is a highlight of the location to show in more detail the abnormal (mispositioned) pixel. (*C*) ALIGNOGRAM screen (in blue with white dashed rectangle) shows in the yellow dotted ellipse, the quantitative measurement of the disorganization created by the misarranged pixel. This is obvious as compared to Fig. 2*B*.

Figure 3*A* shows the same femoral shaft as in Fig. 2*A*. However, in this femur, a single pixel of attenuation value of 220 has been modified in such a way that it is now by virtue of its location and attenuation value, out‐of‐place (ie, mispositioned, abnormal), thereby producing local disorganization or misarrangement. Figure 3*B* is a highlighted location of the bone which shows in more detail, the single mispositioned (misarranged) pixel. The corresponding disorganization curve is shown in Fig. 3*C*. As seen in the yellow dotted circle, the disorganization created by the misarranged pixel is captured by an abrupt peak in DV on the curve. The DV at the location of the misarranged pixel is 27.2 compared to 3.29 when there was no misarranged pixel at that location (comparing Figs. 2*C* and 3*C*). The corresponding CDV was 6.15 without a misarranged pixel versus 8.79 in the presence of a misarranged pixel (solid yellow ellipses).

**Fig. 4:**
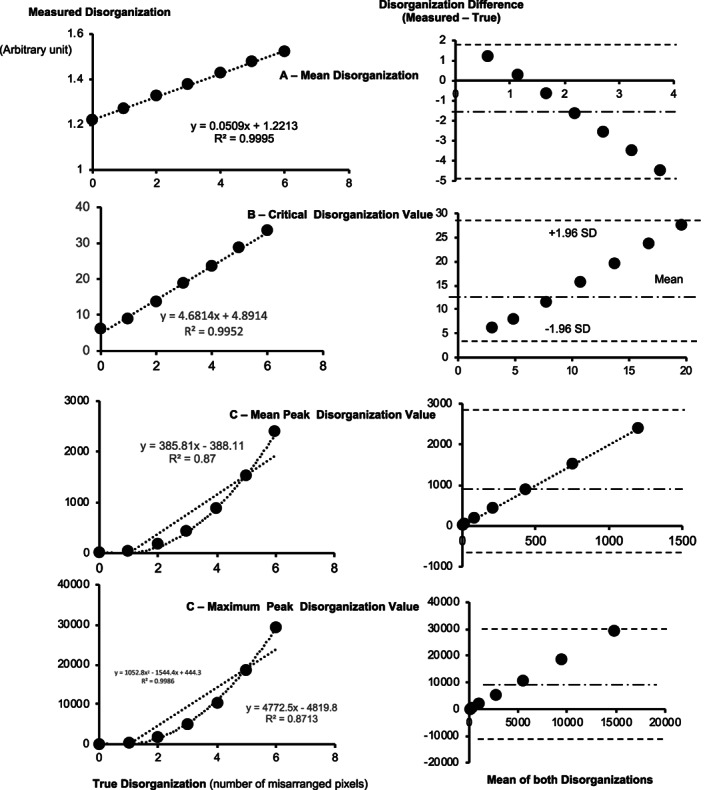
Left panels: Correlations between the measured disorganization and the gold standard (true disorganization) for the mean disorganization of the femoral shaft (*A*), the critical disorganization value (*B*), the mean peak disorganization (*C*), and the maximum peak disorganization (*D*). These correlations were obtained by mispositioning pixels of attenuation of 220. The number of misarranged pixels is on the *x*‐axis, and the measured disorganization metrics are on the *y*‐axis. Right panels: corresponding Bland‐Altman plots for measured disorganization metrics. All values fall within the limits of the agreement.

As shown in Fig. 4, the extent of disorganization as measured by the method (ALIGNOGRAM) predicted the actual or true disorganization. The greater the number of mispositioned pixels (true disorganization), the greater the metrics of disorganization such as the mean DV, CDV, mean PDV (Peak Disorganization Value), and maximum PDV (Peak Disorganization Value) with *R* ^2^ ranging from 0.87 to 0.99 (Fig. 4*A*–*D*, left panels). Bland‐Altman plots showed that there was an agreement between the measurements; with all values falling within the limits of the agreement. The measured disorganization was higher than the created (true) disorganization (Fig. 4*A*–*D*, right panels).

The ability of the metrics of disorganization to predict true disorganization obtained by mispositioning pixels of attenuation of 220, was reproduced for pixels with varying attenuation values. Examples for attenuations of zero (0) and 165 are shown respectively in Figs. S2 and S3 in the supplementary material section. An exemplary illustration of the ability of the method to detect disorganization produced by pixels of high attenuation value is provided in the supplementary material section (Fig. S4). Correlations for these attenuation values between true and measured disorganization had *R* ^2^ values ranging from 0.84 to 0.99. Bland‐Altman plots further showed that there was good prediction between measurements with all values within the limits of the agreements (Figs. S1 and S2).

### Accuracy of disorganization quantification—in vivo clinical verification/validation

**Fig. 5:**
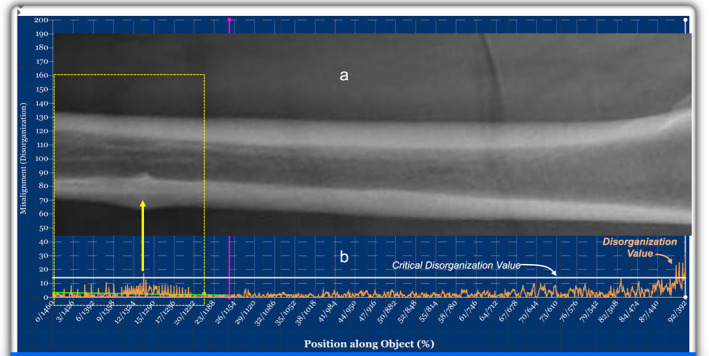
(*A*) X‐ray of the femur of a patient on long‐time antiresorptive therapies. The cortical thickening and endosteal protrusion of the cortex are visible (yellow dotted square). (*B*) shows the corresponding ALIGNOGRAM. As compared to Fig. 2 (ALIGNOGRAM of a normal femur), the disorganization curve (orange) is irregular, with high disorganization values with most exceeding the expected disorganization value for that portion (green line). A peak curve corresponding to the endosteal protrusion is visible (yellow arrow) and exceeds both the green and white lines. Illustration of the ability of disorganization measurement (ALIGNOGRAM) to identify the early stage of atypical femoral fractures.

Disorganization curves (ALIGNOGRAMs) of the femoral shaft of patients with various stages of bone abnormalities associated with AFFs agreed well with actual (true) disorganization features seen on X‐ray images. Exemplary illustrations are shown in Figs. 5 and S6 with additional demonstrations provided in the supplementary material section (Fig. S5).

As compared with Fig. 1 (ALIGNOGRAM of a normal shaft), Fig. 5*A* shows an X‐ray image of the femoral shaft with locally disorganized cortical and medullary space producing endosteal and periosteal thickening (yellow dotted square) in a patient on long‐term antiresorptive therapy. The corresponding portion of the ALIGNOGRAM curve shows an irregular, chaotic pattern with higher disorganization values. As compared with Fig. 1 (normal ALIGNOGRAM) the relatively flat and regular curve with low DVs at the distal end of the shaft has been lost. The measured CDV (14.94) is more than twice the value observed in a normal shaft (6.15). Noticeably, there is a sharp spike on the curve at the location corresponding to the most protruded part of the thickened cortex (yellow arrow).

#### The extent of disorganization in fracture‐free femurs and AFFs

Women with AFFs and fracture‐free controls were of similar age (70.9 ± 1.82 versus 68.04 ± 1.83; *p* = 0.38).

The mean DV in fracture‐free femurs of age‐matched controls was 1.85 ± 0.11; hence, higher in fracture‐free femurs contralateral to one with AFFs (3.76 ± 0.59, *p* < 0.0001 relative controls), and highest in femurs of women with AFFs (19.62 ± 2.83, *p* < 0.0001 relative the two other groups).

**Fig. 6:**
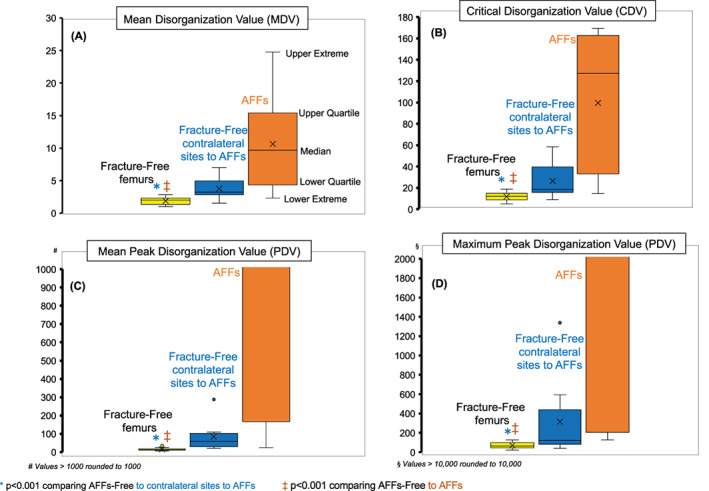
Box and whisker plots showing disorganization values in fracture‐free femurs from age‐matched peers (controls) (yellow color), fracture‐free femurs contralateral to with AFFs (blue), contralateral AFFs femurs (orange). The extent of disorganization was lowest in the control's femurs, higher on the contralateral femurs to the AFFs, and highest on AFFs femurs. This was seen with the mean disorganization values (*A*), critical disorganization values (*B*), mean peak disorganization values (*C*), and maximum peak disorganization values (*D*). Quantification of the mean disorganization value (MDV), critical disorganization value (CDV), and peak disorganization values (mean and maximum) may allow early identification of patients at risk for atypical femoral fractures as shown in Fig A‐D.

Similar results were observed for the CDV (11.87 ± 0.84 versus 26.41 ± 5.87 versus 99.63 ± 21.87, respectively, all *p* < 0.001), mean PDV (14.84 ± 1.27 versus 84.73 ± 27.3 versus 2998.86 ± 1253, all *p* < 0.0001), maximum PDV (67.95 ± 6.34 versus 317.49 ± 139.51 versus 14208.53 ± 6679; all *p* < 0.0001) (Fig. 6).

#### Precision

The precision errors for disorganization quantification expressed as root mean square coefficient of variation (RMSCV were 1.72% for the average DV; 2.16% for the CDV, 3.58% for the Mean PDV; and 4.69% for Maximum PDV.

## Discussion

We report for the first time a quantification method for disorganized (misaligned, disarranged) bone components from X‐ray images. The method accurately and reproducibly detects disorganization within the bone and quantifies the extent or magnitude of this disarrangement.

The method lies in defining an orientation target point. If all pixels forming the object (eg, bone) have the same orientation (or alignment, direction) vis‐à‐vis this target point, then these pixels are co‐aligned, and well arranged in relation to each other and the object or bone is well organized. Hence, conversely, if pixels forming the object have different orientations with respect to the target point, then these pixels are misaligned or disorganized. The greater, and the more random the differences in the orientations of the pixels forming an object, the greater the disorganization of the object (eg, bone in this context).

The measured disorganization showed an excellent correlation with the true disorganization (“gold standard”) created by disarranging pixels with an *R* ^2^ > 0.84. Although the linear model was used to assess the correlation, it was apparent that the best‐fitted relationship true versus measured disorganization was an exponential model. This suggests that the greater the true disorganization, the greater the ability of the method to detect the disorganization. An excellent agreement between methods was seen on the Bland‐Altman plots with nearly all values falling within the limits of the agreement. Further, in vivo testing showed that the method had a very good ability to identify disorganization created by bone abnormalities such as those preceding or leading to AFFs. This includes abnormal, disorganized cortical and endosteal thickening, incomplete AFFs, or complete AFFs.

The robustness of the method was further demonstrated by its ability to identify and quantify disorganization as small as that produced by one single misplaced pixel on an X‐ray image. During in vivo testing, good organization of bone at the distal end of the femoral shaft of a healthy femur was accurately captured by the relatively flat disorganization curve with low values (typically <10). In addition, the gradual, gentle angulation of the shaft proximally to join the femoral neck and the ensuing normal (physiological) rearrangement of bone tissue required for this process were captured by a gradual increase of disorganization values, which correctly peaked at the lesser trochanter.

The clinical usefulness of the method was further demonstrated by showing its ability to distinguish fracture‐free femurs from those with AFFs. Bilaterality is an important feature of AFFs with about 30% also affecting the contralateral femur.^(^ ^9^, ^17^ ^)^ Hence, the ability of the disorganization measurement to distinguish the contralateral femurs of AFFs from other normal femurs supports the view that disorganization plays a role in the occurrence of AFFs. Furthermore, it highlights the potential clinical utility of the proposed method.

The mechanism via which disorganization (misplacement, disarrangement) produces abnormality, instability, and structural failure (collapse) is well exemplified by the collapse of the Jenga tower. In this game, if a block is placed at the wrong position, or pulled out (removed) from where it should be, as a result of the disorganization so produced, the transfer of loads between components (blocks) will become abnormal, the tower will be become unstable, shaky, and collapse. It should be noted that the instability or collapse of the Jenga tower is not an issue of mass or density. This is also not a problem of decay because blocks are all individually normal. It is problem of the wrong block, at the wrong place leading to disorganization.

Disorganization may produce bone diseases via a mechanism similar to the one leading to instability, and ultimately the collapse of the Jenga Tower. In brief, as recently described, disorganization (misalignment, disarrangement) leads to an ineffective (abnormal) transfer or conduction of loads (forces) between bone components. As a result, the damage is produced triggering inflammation and a series of biological and anatomopathological events that lead to bone abnormalities, bone fragility, and ultimately fractures.^(^ ^22^, ^23^ ^)^

A good organization requires the “*right amount of bone component, at the right place*”. A *disorganized component is a component where it should not be* (ie, at the incorrect location). Hence, the concept of disorganization should be clearly distinguished from mass (ie, the amount of material). Mass relates to one single structure and contains little to no information about the spatial position or the geometry of the structure. A structure of any mass (high or low) may if at the wrong place, be a misfit and hence lead to disorganization, instability, and ultimately the collapse of the entire object. A component with high mass (if misplaced) may produce severe disorganization as shown in Fig. 3. A high attenuation (surrogate of high mass) may be due to many causes such as localized hypermineralized foci, sclerotic lesions, or cortical thickening. These features have been associated with fragility fractures such as AFFs, stress fractures, or fractures occurring in many metabolic and genetic diseases such as hypophosphatasia, CKD‐MBD, or osteopetrosis.^(^ ^9^, ^13^, ^14^ ^)^ Although the mechanism by which fragility occurs in these diseases remains unclear, it is likely that this may be due to disorganization so produced.

Similarly, a structure of low mass if misplaced, may be a misfit and cause disorganization of the whole object. A practical example is a low mass (attenuation) produced by the cortical breach due to AFFs causing severe disorganization shown in Fig. S6. This was further demonstrated in the supplementary material (Figs. S2 and S5). From a clinical perspective, low attenuation pixels producing bone disorganization could have many pathological causes. This includes, for example, porosity, abscesses, infarcts, or necrosis.

Conversely, a low attenuation pixel if correctly positioned (eg, in such a way that its “fits” well other constituents) may not cause any disorganization. This also has many implications. As an example, this may explain, at least partly, the variable and inconsistent relationship between porosity (a low attenuation feature) and fracture and risk. Porosity in young adulthood is due to Haversian canals. It is physiological and not associated with fracture risk. We propose that this is because this porosity is well organized. On the contrary, porosity in old age produced by remodeling imbalance is disorganized and shows a greater association with fracture risk.^(^ ^24^ ^)^ We propose that the contribution of porosity to fracture risk may depend not only on the decay its produces but on its ability to produce disorganization.

The view that disorganization is an independent biomarker of bone abnormalities and fractures has important implications for the pathogenesis of bone diseases. The current paradigm is that the higher the amount of bone mass (or BMD), the better the bone. Hence, high BMD is viewed as a marker of low fracture risk.^(^ ^1^, ^2^, ^3^, ^4^ ^)^ Measurement of disorganization is a significant change from this concept. A high‐density structure (eg, a locally high BMD) may be detrimental if its produces disorganization. Furthermore, a low density (or low attenuation) structure usually viewed as disadvantageous may in fact be good for the overall organization of the object (eg, bone). As stated above as an example, a well‐organized porosity although associated with overall low‐density contrary to the current view, may not be abnormal or a sign of a diseased bone. These differences and complementarities between organization and BMD make the quantification of disorganization as proposed here, a potentially complementary tool in the assessment of bone health.

The concept of bone disorganization should also be distinguished from structural decay. Decay is a paradigm that implies “unhealthy” or deteriorated components. In the context of bone, deterioration is usually produced by remodeling imbalance resulting in loss of bone on its surfaces.^(^ ^18^ ^)^ In contrast, disorganization relates to misassembled or improperly stacked components. This may occur regardless of whether these components are individually healthy or decayed. Bone disorganization can even be severe, yet individual elements are healthy (ie, no decay and normal mass). In the case of bone, as shown in Fig. 3, for example, high attenuation pixels simulating high mineralized bone matrix produced marked disorganization yet did not cause structural deterioration (eg, cortical thinning or porosity). As another example, in clinical settings, cortical thickening with endosteal protrusion as shown in Fig. 5 creates disorganization even though there may be no decay (no porosity, no cortical thinning).

From a diagnostic perspective, current tools available for the assessment of bone health and monitoring of the effects of treatments or diseases rely on two main approaches: (i) measures of bone mass (and its derivatives) using DXA or similar instruments^(^ ^2^ ^)^; and (ii) measures of structural decay using QCT or the more advanced HR‐pQCT.^(^ ^2^, ^4^ ^)^ As explained above, neither tools or approaches are designed or intended to be used for the quantification of disorganization—a different bone property. Hence, we propose that a method of quantification of disorganized bone is a missing tool in the diagnostic arsenal of scientists and clinicians. As an example of clinical application, bone abnormalities that produce disorganization such as abscesses, necrosis, or infarct may be suitably detected by quantifying the disorganization. This is an application in bone health assessment beyond the current paradigm centered on fracture risk assessment. In addition, there are many bone diseases associated with increased fracture risk but normal to high density, little to no decay. This makes these diseases challenging to assess and enigmatic because they are poorly correlated with measures of density such as BMD or bone structure measured using QCT or HRpQCT.^(^ ^2^, ^10^, ^25^ ^)^ However, it should be noted that these diseases often share in common their association with disorganized bone, presenting with features such as thick structures (sclerosis, high mineralization), deformities, bowing, or areas of necrosis. This includes high bone mass diseases or metabolic and genetic disorders such as osteopetrosis, hypophosphatasia, osteogenesis imperfecta, glucocorticoid therapy, and CKD‐MBD.^(^ ^10^, ^14^, ^26^ ^)^ Notably, AFFs as we propose, are one example of this category of diseases—these fractures are associated with normal or mildly reduced BMD, and there is no evidence of structural decay.^(^ ^5^ ^)^ Imaging shows thick cortices and disorganized bone with examples shown in Fig. 5, and Figs. S5 and S6. We propose that quantification of disorganization provides a reliable tool for the assessment of these diseases. Potential applications are enormous because of the large family of diseases that share these characteristics. We have referred to these as disorders of disorganized bone tissue.^(^ ^22^, ^23^ ^)^

The method is here described in the context of X‐rays images, which are widely available. Hence, disorganization measurement can be readily and easily incorporated into both research and clinical practice. The maximum pixel size and resolution required for the assessment of disorganization bone are that of standard X‐rays. This is generally 175 μm or less.^(^ ^15^ ^)^ This is like the resolution of X‐ray images used in this study. Hence, a similar higher resolution (smaller pixel size) should be sufficient for the use of the method. Furthermore, the method can be used for any other type of 2D image (photographs or scanning electron microscopic images). The method may also be used to quantify disorganization in 3D images such as QCT, HR‐pQCT, or magnetic resonance imaging (MRI). For 3D images, quantification and production of the disorganization curve (ALIGNOGRAM) is done in the *xyz* axis. Applications to other imaging modalities will be the subject of future work.

The method is described in the setting of the measurement of the disorganization of the femoral shaft. However, the same method can be used to quantify the disorganization of any bone, any material, structure, tissue, or object. All that is required is the definition of a different orientation target point in lieu of the middle of the acetabular weight‐bearing dome used for the femoral shaft. These potential applications to the bones will also be the subject of future work.

The method has several limitations. It quantifies the degree of disarrangement of bone pixels and this regardless of the cause (etiology) of this disorganization. The method does not, and is not intended to, diagnose the cause of the disorganization (eg, due to defective assembly and/or configuration of protein such as collagen type I in osteogenesis imperfecta, or disorganized mineralized matrix).^(^ ^11^, ^22^, ^23^ ^)^ The interpretation of disorganization depends on the clinical context. This is similar to BMD, which provides a quantification of the amount of bone mass per unit area. The etiology of a high or low BMD then depends on the clinical context. Bone disorganization measured is that of pixels in relation to each other rather than direct bone tissue disorganization. However, pixel attenuation values reflect tissue composition (subject to the limits of the imaging device such as pixel size and resolution). It should be noted that the same assumption applies to all imaging‐based assessments. There are no other methods of quantification of disorganization for comparisons. This report is limited to the description and validation of a method of quantification of disorganization. The mechanism via which disorganization may produce bone abnormalities and fragility has been described.^(^ ^22^, ^23^ ^)^ It is anticipated that this will drive further work in this area.

An advantage is that the proposed method only uses the attenuation value of pixels. This is an important difference from other approaches that measure density (and its derivatives) and hence, requires an additional step of calibration with all the challenges associated with this process. This quantification method for bone disorganization does not require calibration.

In conclusion, we report the first‐ever method to quantify bone disorganization. The method is accurate and reproducible. Furthermore, it is robust and highly sensitive to the presence of disorganization. As an example, it could detect disorganization produced by a single pixel. Disorganization refers to improper stacking or assembly of components that form an object. Hence, this feature is fundamentally different from other commonly measured bone properties such as the amount of bone (eg, BMD) or indicators of structural decay measured using tools such as QCT, and the more advanced HR‐pQCT.

Measurement of disorganization may have numerous potential applications. The contribution of disorganization to bone diseases is unstudied even though many bone diseases are known to be associated with disorganized bone tissue; and disorganized bone pixels (components) on X‐ray images. These include hypophosphatasia, osteogenesis imperfecta, glucocorticoid therapy, diabetes mellitus, CKD‐MBD, and AFF or other stress fractures.^(^ ^9^, ^10^, ^11^, ^12^, ^13^, ^14^ ^)^ This novel tool, by bringing the measurement of bone disorganization into routine clinical and research settings, has the potential to revolutionize our understanding of bone in health and disease. Besides being potentially a necessary complementary measurement to bone mass or structural decay, quantification of disorganization may allow better insights into pathogenesis, diagnosis, and treatment of bone diseases.

## Author Contributions

**Catherine Shore‐Lorenti:** Investigation; methodology; validation; writing – original draft; writing – review and editing. **Hanh H Nguyen:** Conceptualization; investigation; methodology; validation. **Cherie Chiang:** Investigation; methodology; validation; visualization. **Frances Milat:** Investigation; methodology; supervision; validation; writing – original draft; writing – review and editing. **Peter R Ebeling:** Conceptualization; funding acquisition; investigation; methodology; project administration; supervision; validation; visualization; writing – original draft; writing – review and editing.

### Peer Review

The peer review history for this article is available at https://publons.com/publon/10.1002/jbm4.10713.

## Supporting information


**Fig. S1.** Left panels: Correlations between the measured disorganization and the gold standard (true disorganization) for the mean disorganization of the femoral shaft (*A*), the critical disorganization value (*B*), the mean peak disorganization (*C*), and the maximum peak disorganization. These correlations were obtained by mispositioning pixels of attenuation of zero (0). The number of misarranged pixels is on the *x*‐axis, and the measured disorganization metrics are on the y‐axis. Right panels: Corresponding Bland and Altman plots for measured disorganization metrics. All values fall within the limits of the agreement.

**Fig. S2.** (*A*) is an X‐ray image of the femur of a healthy woman. The same femur as in Fig. 1 (main manuscript). In this femur, 4 pixels of attenuation value 0 (zero) have been misarranged to produce disorganization (dark pixels in the yellow rectangle with attenuation of zero). (*B*) is a highlight of the location to show in more detail the abnormal (mispositioned) pixels. (*C*) Shows in the yellow dotted circle, the quantitative measurement of the disorganization created by the misarranged pixels; This sudden peak corresponding to the created disorganization is obvious.

**Fig. S3.** Left panels: Correlations between the measured disorganization and the gold standard (true disorganization) for the mean disorganization of the femoral shaft (*A*), the critical disorganization value (*B*), the mean peak disorganization (*C*), and the maximum peak disorganization. These correlations were obtained by mispositioning pixels of attenuation of 165. The number of misarranged pixels is on the *x*‐axis, and the measured disorganization metrics are on the *y*‐axis. Right panels: Corresponding Bland and Altman plots for measured disorganization metrics. All values fall within the limits of the agreement.

**Fig. S4.** (*A*) is an X‐ray image of the femur of healthy woman. The same femur as in Fig. 1 (main manuscript). In this femur, 4 pixels of the attenuation value 165 have been disarranged to produce disorganization (dark pixels in the yellow rectangle with attenuation of 165). (*B*) is a highlight of the location to show in more detail the abnormal (mispositioned) pixels. (*C*) Shows in the yellow dotted circle, the quantitative measurement of the disorganization created by the misarranged pixels; This sudden peak corresponding to the created disorganization is obvious. The ability of the Alignogram to identify the disorganization produced by a bone component of high density or attenuation.

**Fig. S5.** (*A*) is an X‐ray of the femur of a patient on long‐time antiresorptives therapy. The atypical femoral fractures with the transverse and minimal comminution are features (Yellow dotted square). (*B*) shows the corresponding ALIGNOGRAM. The disorganization is very irregular, haphazard, chaotic, and very distinct from a normal ALIGNOGRAM (Figure 1, main manuscript). There are prominent peaks on the curve at the location corresponding to the location of the displaced fracture fragments. These peaks are abrupt and needle‐like, nearly vertical. This is consistent with the transverse character of the disorganization created by the fracture (*Yellow dotted square*). At other locations of the curve, disorganization values are much higher than observed in a normal femur.

**Fig. S6.** (*A*) shows an X‐ray of the femoral shaft of a patient with an incomplete AFF resulting in a sharp local cortical break producing disarrangement (disorganization) (*yellow arrow*). Adjacent to the sharp transcortical disorganization due to the incomplete fracture, there is also an area of misarranged bone tissue involving both the marrow cavity and the cortex (*Yellow dotted square*). As seen in Fig. 6*B*, the measured disorganization accurately captured the incomplete fracture and displayed it as a sudden, abrupt peak on the curve (*Orange peaks on the curve matching the corresponding yellow arrow*). Furthermore, the curve also shows an area with irregular and higher disorganization values corresponding to the corticomedullary reactions adjacent to incomplete fracture on the X‐ray image (*Yellow dotted square*).

## Data Availability

Data are available on approval of an appropriate application to Transcontinental Atypical Femoral Fracture Consortium (TRAFFIC) study committee. Further information is available from https://trafficstudygroup.org.

## References

[1] Siris ES , Miller PD , Barrett‐Connor E , et al. Identification and fracture outcomes of undiagnosed low bone mineral density in postmenopausal women: results from the National Osteoporosis Risk Assessment. JAMA. 2001;286:2815‐2822. 11735756 10.1001/jama.286.22.2815

[2] Choksi P , Jepsen KJ , Clines GA . The challenges of diagnosing osteoporosis and the limitations of currently available tools. Clin Diabetes Endocrinol. 2018;4:12. 29862042 10.1186/s40842-018-0062-7 PMC5975657

[3] Keaveny TM , Hoffmann PF , Singh M , et al. Femoral bone strength and its relation to cortical and trabecular changes after treatment with PTH, alendronate, and their combination as assessed by finite element analysis of quantitative CT scans. J Bone Miner Res. 2008;23(12):1974‐1982. 18684084 10.1359/JBMR.080805 PMC2686921

[4] Samelson EJ , Broe KE , Xu H , et al. Cortical and trabecular bone microarchitecture as an independent predictor of incident fracture risk in older women and men in the bone microarchitecture international consortium (BoMIC): a prospective study. Lancet Diabetes Endocrinol. 2019;7:34‐43. 30503163 10.1016/S2213-8587(18)30308-5 PMC6354581

[5] Zanchetta MB , Diehl M , Buttazzoni M , et al. Assessment of bone microarchitecture in postmenopausal women on long‐term bisphosphonate therapy with atypical fractures of the femur. J Bone Miner Res. 2014;29(4):999‐1004. 24115250 10.1002/jbmr.2107

[6] Kanis JA . Diagnosis of osteoporosis and assessment of fracture risk. Lancet. 2002;359(9321):1929‐1936. 12057569 10.1016/S0140-6736(02)08761-5

[7] Black DM , Bauer DC , Vittinghoff E , et al. Foundation for the National Institutes of Health bone quality project. Treatment‐related changes in bone mineral density as a surrogate biomarker for fracture risk reduction: meta‐regression analyses of individual patient data from multiple randomised controlled trials. Lancet Diabetes Endocrinol. 2020;8(8):672‐682. 32707115 10.1016/S2213-8587(20)30159-5

[8] Hernandez CJ , van der Meulen MC . Understanding bone strength is not enough. J Bone Miner Res. 2017;32(6):1157‐1162. 28067411 10.1002/jbmr.3078 PMC5466476

[9] Shane E , Burr D , Abrahamsen B , et al. Atypical subtrochanteric and diaphyseal femoral fractures: second report of a task force of the American Society for Bone and Mineral Research. J Bone Miner Res. 2014;29(1):1‐23. 23712442 10.1002/jbmr.1998

[10] Qian Q . Inflammation: a key contributor to the genesis and progression of chronic kidney disease. Contrib Nephrol. 2017;191:72‐83. 28910792 10.1159/000479257

[11] Nijhuis WH , Eastwood DM , Allgrove J , et al. Current concepts in osteogenesis imperfecta: bone structure, biomechanics and medical management. J Child Orthop. 2019;13(1):1‐11. 30838070 10.1302/1863-2548.13.180190 PMC6376438

[12] Rasmussen NH , Dal J , de Vries F , van den Bergh JP , Jensen MH , Vestergaard P . Diabetes and fractures: new evidence of atypical femoral fractures? Osteoporos Int. 2020;31(3):447‐455. 31838553 10.1007/s00198-019-05224-y

[13] Milgram JW , Jasty M . Osteopetrosis: a morphological study of 21 cases. J Bone Jt Surg Am. 1982;64:912‐929. 7085720

[14] Villa‐Suárez JM , García‐Fontana C , Andújar‐Vera F , et al. Hypophosphatasia: a unique disorder of bone mineralization. Int J Mol Sci. 2021;22(9):4303. 33919113 10.3390/ijms22094303 PMC8122659

[15] Huda W , Abrahams RB . X‐ray‐based medical imaging and resolution. AJR Am J Roentgenol. 2015;204(4):W393‐W397. 25794088 10.2214/AJR.14.13126

[16] Lin EC . Radiation risk from medical imaging. Mayo Clin Proc. 2010;85:1142‐1146. 21123642 10.4065/mcp.2010.0260 PMC2996147

[17] Nguyen HH , Lakhani A , Shore‐Lorenti C , et al. Asian ethnicity is associated with atypical femur fractures in an Australian population study. Bone. 2020;135:115319. 32179169 10.1016/j.bone.2020.115319

[18] Glaseby CA . An analysis of histogram‐based thresholding algorithms. Graph Models Im Proc. 1993;55:532‐537.

[19] Bowman KF Jr , Fox J , Sekiya JK . A clinically relevant review of hip biomechanics. Arthroscopy. 2010;26(8):1118‐1129. 20678712 10.1016/j.arthro.2010.01.027

[20] Singhal V , Bredella MA . Marrow adipose tissue imaging in humans. Bone. 2019;118:69‐76. 29331301 10.1016/j.bone.2018.01.009 PMC6039291

[21] Zebaze R , Ghasem‐Zadeh A , Mbala A , Seeman E . A new method of segmentation of compact‐appearing, transitional and trabecular compartments and quantification of cortical porosity from high resolution peripheral quantitative computed tomographic images. Bone. 2013;54(1):8‐20. 23334082 10.1016/j.bone.2013.01.007

[22] Zebaze RM , Jones AC , Pandy MG , Knackstedt MA , Seeman A . Differences in the degree of bone tissue mineralization account for little of the differences in tissue elastic properties. Bone. 2011;48:1246‐1251. 21385633 10.1016/j.bone.2011.02.023

[23] Zebaze R , Ebeling PR . Disorganization and musculoskeletal diseases: novel insights into the enigma of unexplained bone abnormalities and fragility fractures. Curr Osteoporos Rep. 10.1007/s11914-022-00759-2. 36494594

[24] Zebaze RM , Ghasem‐Zadeh A , Bohte A , et al. Intracortical remodelling and porosity in the distal radius and post‐mortem femurs of women: a cross‐sectional study. Lancet. 2010;375(9727):1729‐1736. 20472174 10.1016/S0140-6736(10)60320-0

[25] Larsen MS , Schmal H . The enigma of atypical femoral fractures: a summary of current knowledge. EFORT Open Rev. 2018;3(9):494‐500. 30305933 10.1302/2058-5241.3.170070 PMC6174857

[26] Canalis E , Mazziotti G , Giustina A , Bilezikian JP . Glucocorticoid‐induced osteoporosis: pathophysiology and therapy. Osteoporos Int. 2007;18(10):1319‐1328. 17566815 10.1007/s00198-007-0394-0

