# Supplementary material for: A Quantification Method for Disorganized Bone Components: Application to the Femoral Shaft
Source: JBMR Plus. 2023 Jan 3;7(2):e10713. doi: 10.1002/jbm4.10713 (PMC9893270; doi:10.1002/jbm4.10713)

# Figure 1S- Supplementary Material

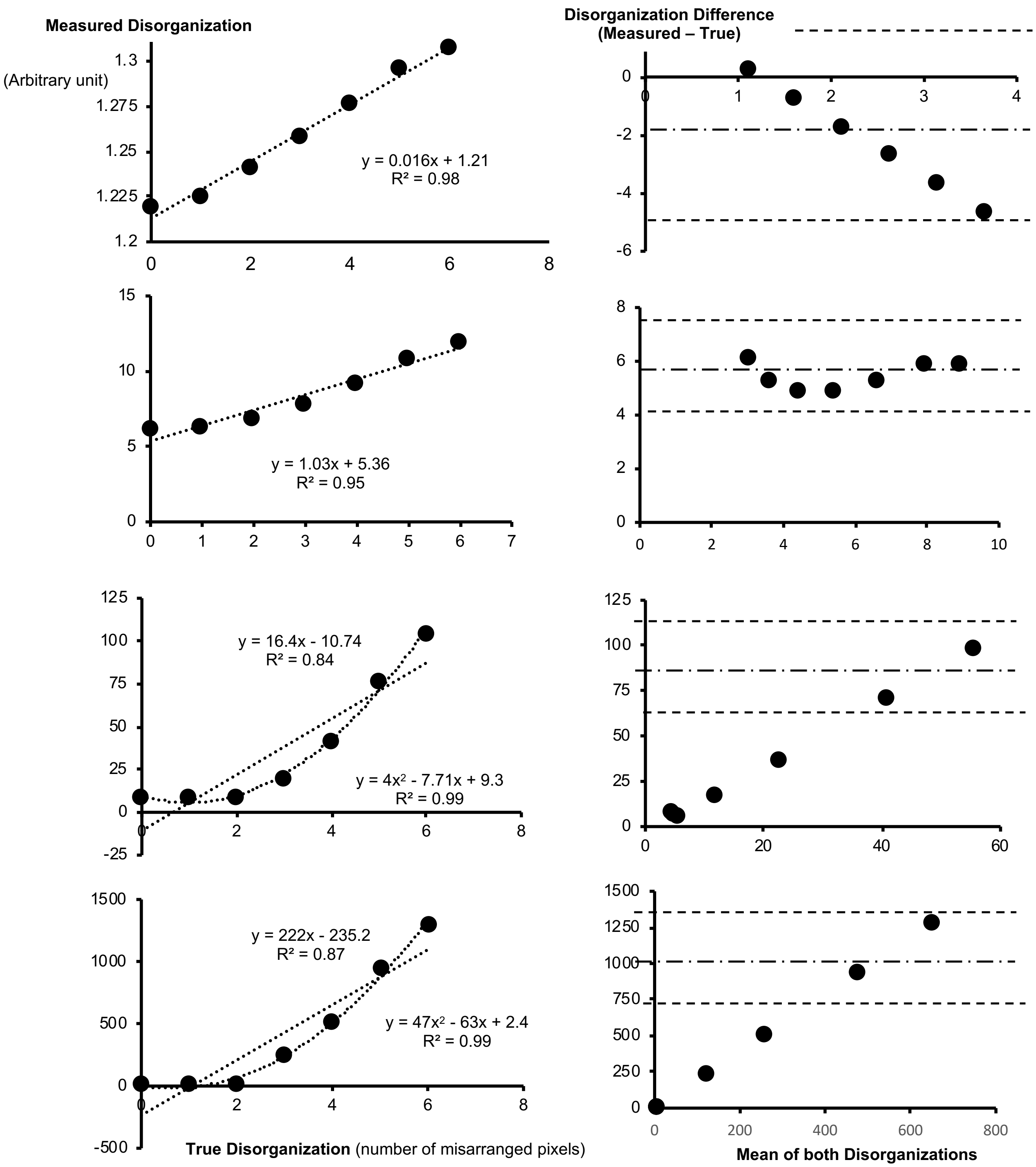

Supplement: Supplementary file 1 — Fig. S1. Left panels: Correlations between the measured disorganization and the gold standard (true disorganization) for the mean disorganization of the femoral shaft (A), the critical disorganization value (B), the mean peak disorganization (C), and the maximum peak disorganization. These correlations were obtained by mispositioning pixels of attenuation of zero (0). The number of misarranged pixels is on the x‐axis, and the measured disorganization metrics are on the y‐axis. Right panels: Corresponding Bland and Altman plots for measured disorganization metrics. All values fall within the limits of the agreement. [file JBM4-7-e10713-s002.pdf]
