# Supplementary figures and images for: A Quantification Method for Disorganized Bone Components: Application to the Femoral Shaft
Source: JBMR Plus. 2023 Jan 3;7(2):e10713. doi: 10.1002/jbm4.10713 (PMC9893270; doi:10.1002/jbm4.10713)

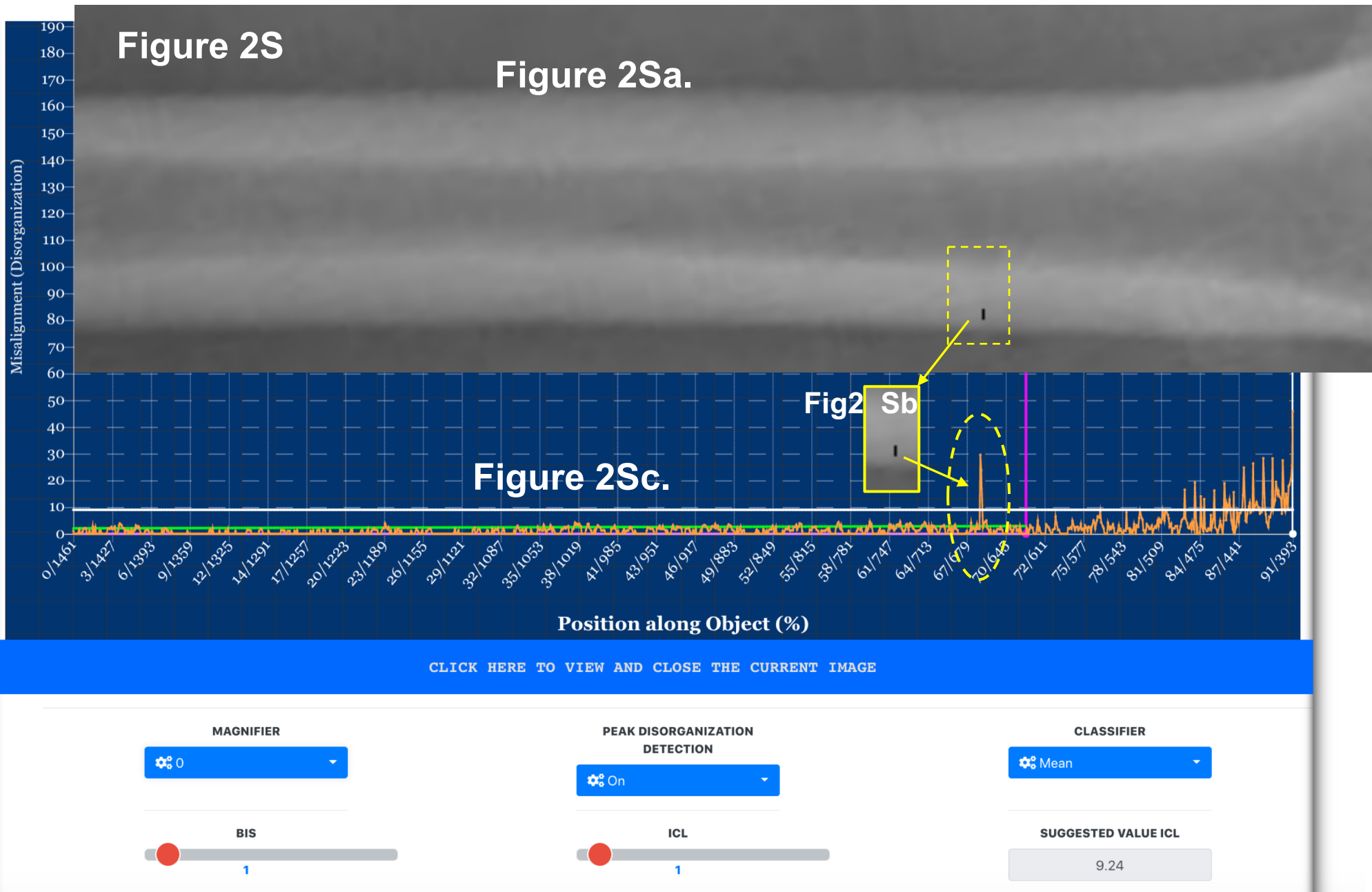

Supplement: Supplementary file 2 — Fig. S2. (A) is an X‐ray image of the femur of a healthy woman. The same femur as in Fig. 1 (main manuscript). In this femur, 4 pixels of attenuation value 0 (zero) have been misarranged to produce disorganization (dark pixels in the yellow rectangle with attenuation of zero). (B) is a highlight of the location to show in more detail the abnormal (mispositioned) pixels. (C) Shows in the yellow dotted circle, the quantitative measurement of the disorganization created by the misarranged pixels; This sudden peak corresponding to the created disorganization is obvious. [file JBM4-7-e10713-s005.pdf]

Figure 5S

Figure 5Sa

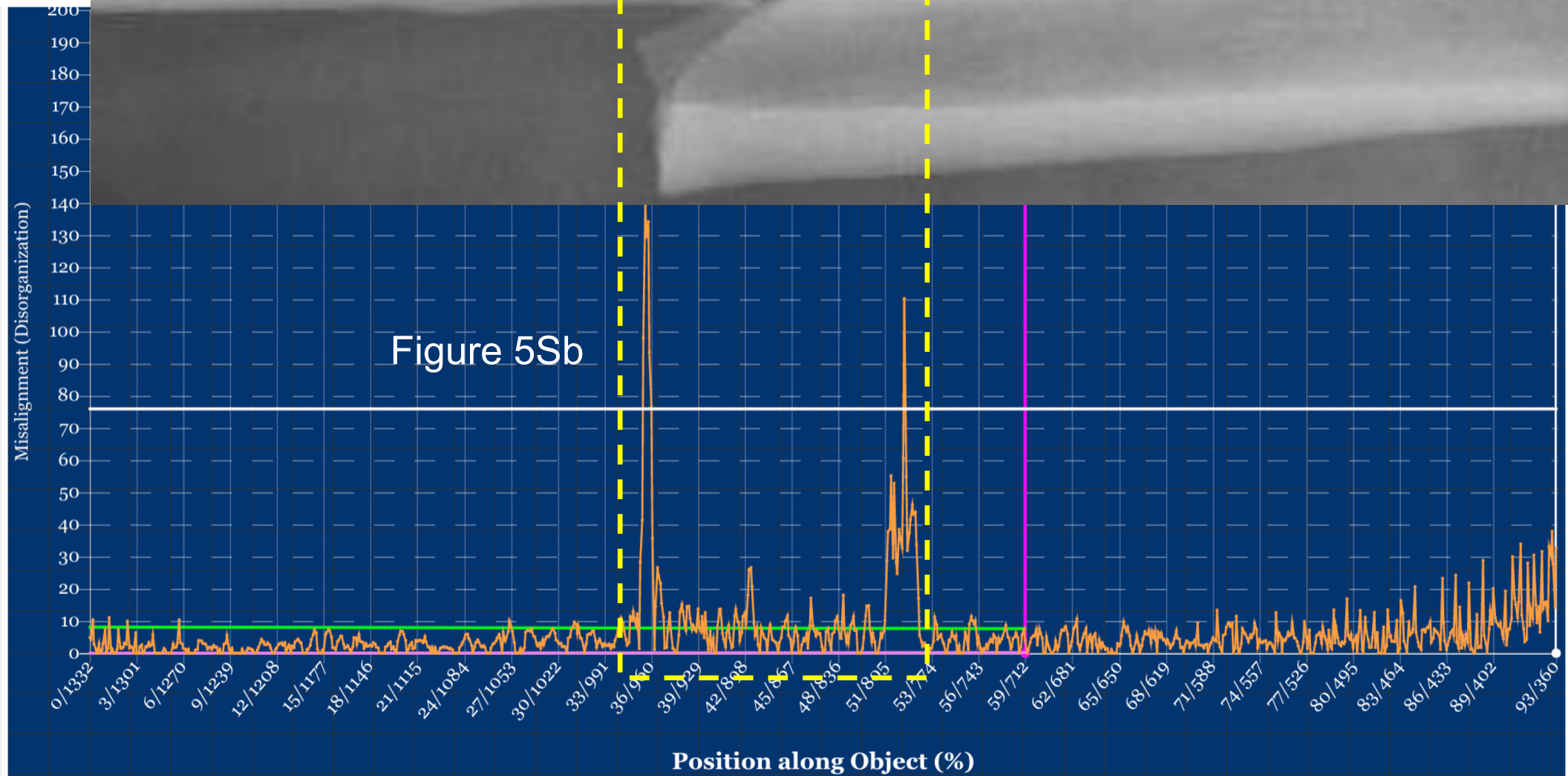

Supplement: Supplementary file 5 — Fig. S5. (A) is an X‐ray of the femur of a patient on long‐time antiresorptives therapy. The atypical femoral fractures with the transverse and minimal comminution are features (Yellow dotted square). (B) shows the corresponding ALIGNOGRAM. The disorganization is very irregular, haphazard, chaotic, and very distinct from a normal ALIGNOGRAM (Figure 1, main manuscript). There are prominent peaks on the curve at the location corresponding to the location of the displaced fracture fragments. These peaks are abrupt and needle‐like, nearly vertical. This is consistent with the transverse character of the disorganization created by the fracture (Yellow dotted square). At other locations of the curve, disorganization values are much higher than observed in a normal femur. [file JBM4-7-e10713-s006.pdf]

Figure 6S

Figure 6Sa

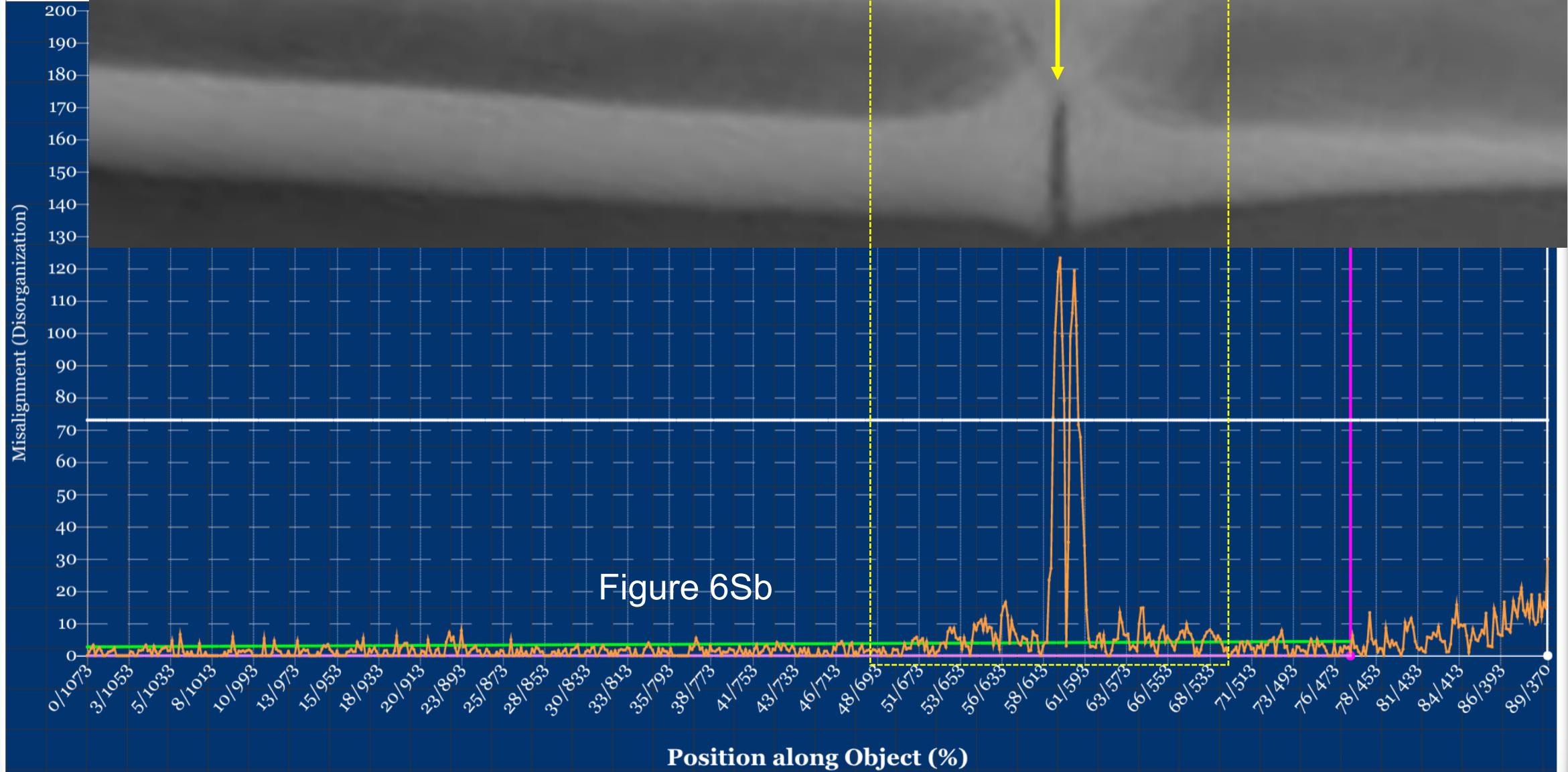

Supplement: Supplementary file 6 — Fig. S6. (A) shows an X‐ray of the femoral shaft of a patient with an incomplete AFF resulting in a sharp local cortical break producing disarrangement (disorganization) (yellow arrow). Adjacent to the sharp transcortical disorganization due to the incomplete fracture, there is also an area of misarranged bone tissue involving both the marrow cavity and the cortex (Yellow dotted square). As seen in Fig. 6B , the measured disorganization accurately captured the incomplete fracture and displayed it as a sudden, abrupt peak on the curve (Orange peaks on the curve matching the corresponding yellow arrow). Furthermore, the curve also shows an area with irregular and higher disorganization values corresponding to the corticomedullary reactions adjacent to incomplete fracture on the X‐ray image (Yellow dotted square). [file JBM4-7-e10713-s001.pdf]
