# Supplementary material for: A Quantification Method for Disorganized Bone Components: Application to the Femoral Shaft
Source: JBMR Plus. 2023 Jan 3;7(2):e10713. doi: 10.1002/jbm4.10713 (PMC9893270; doi:10.1002/jbm4.10713)
Supplement: Supplementary file 4 — Fig. S4. (A) is an X‐ray image of the femur of healthy woman. The same femur as in Fig. 1 (main manuscript). In this femur, 4 pixels of the attenuation value 165 have been disarranged to produce disorganization (dark pixels in the yellow rectangle with attenuation of 165). (B) is a highlight of the location to show in more detail the abnormal (mispositioned) pixels. (C) Shows in the yellow dotted circle, the quantitative measurement of the disorganization created by the misarranged pixels; This sudden peak corresponding to the created disorganization is obvious. The ability of the Alignogram to identify the disorganization produced by a bone component of high density or attenuation. [file JBM4-7-e10713-s004.pdf]

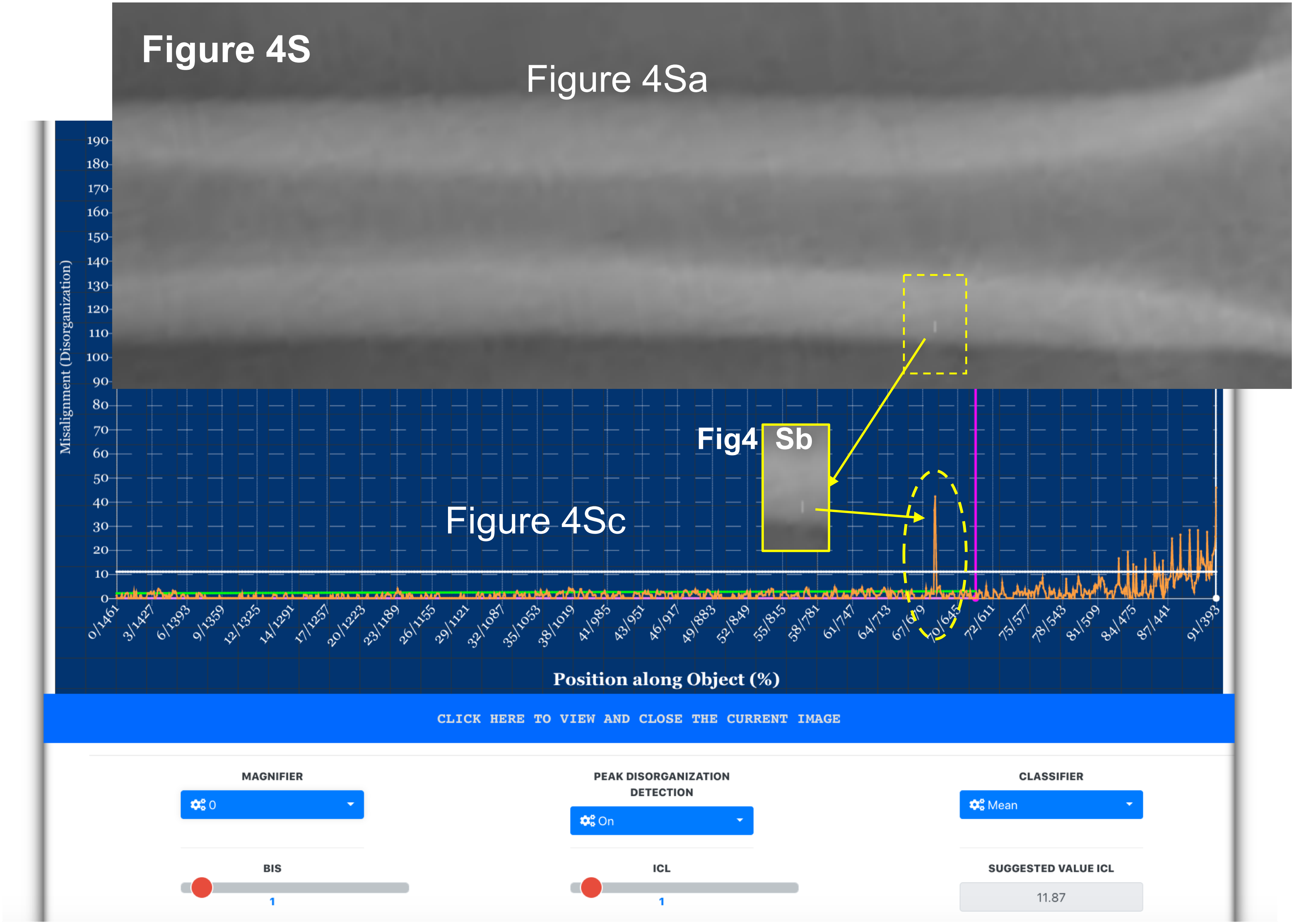

Figure 4S
Figure 4Sa
Fig4 Sb
Figure 4Sc
Misalignment (Disorganization)
Position along Object (%)
CLICK HERE TO VIEW AND CLOSE THE CURRENT IMAGE
MAGNIFIER
0
PEAK DISORGANIZATION DETECTION
On
CLASSIFIER
Mean
BIS
1
ICL
1
SUGGESTED VALUE ICL
11.87
